# Epidemiology of animal bites and post-exposure vaccination coverage in Davao City, Philippines: A five-year retrospective cohort study, 2019–2023

**DOI:** 10.1016/j.onehlt.2026.101622

**Published:** 2026-10-17

**Authors:** Alfredo A. Hinay, April Joy Parilla-Sarabia, Jennifer Ashley H. Reyes, Mariel Phoebe M. Lopez, Rvin John T. Servillon

**Affiliations:** a College of Medical and Biological Sciences, University of the Immaculate Conception, Davao City, Philippines; b Graduate School Department, University of the Immaculate Conception, Davao City, Philippines

**Keywords:** Rabies, One health, Animal bites, Post-exposure prophylaxis, Epidemiology, Zoonosis, Philippines

## Abstract

Rabies remains a major public health threat in the Philippines, particularly in urban areas such as Davao City, where frequent animal bites from domestic reservoirs increase the transmission risk at the human-animal-environment interface. Detailed epidemiological data on animal bites and post-exposure prophylaxis (PEP) coverage are limited. This One Health-framed retrospective cohort study describes trends in bite incidents, assesses PEP coverage, and identifies gaps in rabies prevention efforts from 2019 to 2023.

Surveillance data from the Davao City Animal Bite Treatment Centers captured 98,442 cases. Dogs caused 63.5% of bites, with a significant upward trend (*p* = 0.027; *τ* = 1.0, 95% CI 0.200–1.800); cat bites (36.4%) rose sharply, indicating an emerging reservoir challenge. Children ≤15 years of age showed a significant increase (*p* = 0.027). The 3rd congressional district had the most pronounced rise across demographics and exposure categories. Tissue culture vaccine coverage remained at 100%, equine rabies immunoglobulin rose from 18.5% to 85.9%, whereas human rabies immunoglobulin remained low post-COVID.

Despite universal TCV access, immunoglobulin gaps limit its control. Rising cat bites and severe exposures demand integrated One Health strategies, enhanced surveillance, animal vaccination, and supply chain strengthening, to achieve zero human rabies deaths by 2030.

## 1 Introduction

Rabies is a fatal zoonotic disease that poses a significant threat to public health in many developing countries, including the Philippines [1], [2] Despite being entirely preventable through timely administration of post-exposure prophylaxis (PEP) and effective animal vaccination programs, rabies remains endemic in certain regions, with Davao City being one of the most affected areas. This urban setting exemplifies One Health challenges, where high human-animal interactions and environmental factors amplify the transmission from domestic reservoirs. The disease is primarily transmitted through bites or scratches from infected animals, with dogs as the primary reservoir [2], [3], [4] The high incidence of animal bites and the associated risk of rabies exposure emphasize the urgent need to better understand exposure patterns across human, animal, and environmental domains and evaluate PEP coverage and effectiveness [5], [6], [7].

Davao City has made considerable efforts to address rabies through its network of Animal Bite Treatment Centers (ABTCs), which provide free PEP to bite victims. However, challenges persist, including the high number of animal bite cases, limited vaccine coverage among animals, and gaps in public awareness regarding rabies prevention. In recent years, thousands of residents have sought PEP following potential rabies exposure, which reflects the scale of this problem [8], [9], [10] Despite these interventions, human rabies cases and fatalities continue to occur, highlighting the need for integrated One Health evaluation and implementation of existing rabies control measures.

Rabies elimination requires a multifaceted One Health approach, including robust surveillance, timely treatment of exposed individuals, mass vaccination of animal reservoirs, and community education [11] In the Philippines, the National Rabies Prevention and Control Program has set ambitious goals to eliminate human rabies by 2030 [12] However, achieving this target requires addressing persistent challenges, such as limited access to vaccines in rural areas, underreporting of animal bite cases, and insufficient public knowledge of rabies prevention. As a major urban center with a high population density and elevated incidence of animal bites, Davao City serves as an essential setting for assessing the epidemiology of bites and PEP vaccination coverage through a One Health lens.

This five-year retrospective cohort study analyzed animal bite incidents and post-exposure vaccination coverage in Davao City, Philippines, from 2019 to 2023. By utilizing data from the city's ABTCs, this study aimed to characterize bite epidemiology, identify temporal trends across demographic groups and congressional districts, and evaluate post-exposure vaccination coverage among bite victims. These findings provide a critical assessment of the effectiveness of the current rabies control system in the Philippines, highlighting its strengths and persistent gaps in vaccine and immunoglobulin coverage. These insights will inform evidence-based enhancements to local and national One Health rabies prevention strategies, ultimately supporting the country's goal of eliminating human rabies-related deaths.

## 2 Methods

### 2.1 Study design and setting

This retrospective cohort study analyzed data collected from the ABTCs across Davao City, Philippines, over a five-year period from January 1, 2019, to December 31, 2023. Davao City, located on the island of Mindanao, the largest city in the Philippines by land area and the third most populous city in the country.

### 2.2 Participants

Data collection involved a two-stage process to ensure comprehensive and reliable data capture. In the first stage, all individuals who sought medical consultation for animal bites or scratches at any ABTC in Davao City during the study period were identified and included. In the second stage, these individual case data were systematically aggregated and compiled into annual reports by the central office coordinating the ABTC network in Davao City. These consolidated annual reports maintained complete and standardized records collected from all the treatment centers across the city. Prior to inclusion in the analysis, the data completeness and consistency within these annual reports were carefully verified by the research team. These steps served to minimize selection bias by ensuring the inclusion of all eligible cases citywide and to reduce information bias using standardized and centralized data sources.

### 2.3 Variables

Demographic data collected included age (categorized as <15 and ≥ 15 years) and sex. Animal exposure details consisted of the type of animal responsible for the bite or scratch, classified as dog, cat, or other animals, along with the date of exposure.

Rabies exposure categories were assigned according to the World Health Organization (WHO) guidelines.

Category II: Nibbling of uncovered skin or minor scratches or abrasions without bleeding.

Category III: Single or multiple transdermal bites or scratches, or contamination of mucous membranes with saliva from animal bites or scratches.

Post-exposure prophylaxis data included the type of rabies biologics administered to each patient: Tissue culture vaccine (TCV), human rabies immunoglobulin (HRIG), and equine rabies immunoglobulin (ERIG).

### 2.4 Data sources and ethical considerations

All data were obtained from centralized annual reports of the Animal Bite Treatment Centers (ABTCs) in Davao City, which routinely aggregate case records from individual centers. The Davao City Health Office authorized access to and use of these reports for this study. Ethical approval for the retrospective secondary analysis of anonymized ABTC data was obtained from the University of the Immaculate Conception Institutional Review Board (protocol code: GS-EX-08-25-0059), and the requirement for informed consent was waived. Anonymized data were handled in accordance with the EU 2016/679 General Data Protection Regulation guidelines and the Data Privacy Act of 2012 (Republic Act No. 10173).

### 2.5 Study size

A total of 98,442 animal bite cases reported citywide over five years were initially extracted from the ABTC annual report. After applying the exclusion criteria, all complete and eligible cases were included in the epidemiological and vaccination coverage analyses.

### 2.6 Statistical analysis

Bite incidents from 2019 to 2023 were analyzed by sex, age group, bite source, and rabies exposure category across the 1st to 3rd congressional districts. The yearly counts and proportions were calculated for each category. Temporal trends in the bite incidence and mortality proportions were assessed using the Mann-Kendall test, considering the years as an ordinal variable. Statistical significance was set at *p* < 0.05 using Stata 14 (StataCorp LP).

Kendall's tau (τ) was used to quantify the direction and strength of monotonic trends, ranging from −1 (strong decreasing trend) to 1 (strong increasing trend). The 95% confidence interval (CI) of τ was reported to indicate precision; a CI that excludes zero suggests a significant temporal trend. This non-parametric approach robustly characterizes trends in bite incidence proportions over time.

## 3 Results

### 3.1 Temporal trends of animal bite incidents in Davao City, Philippines

A total of 98,442 animal-bite cases were reported in Davao City from 2019 to 2023 across three congressional districts. Analysis of these incidents revealed a progressive increase in the number of reported cases over the five-year period (Fig. 1, Supplementary Table S1). Males accounted for 47.8% of the total bites, while females accounted for 52.2%, with both groups showing a positive but statistically non-significant upward trend (*p* = 0.086; *τ* = 0.8 [95% CI: −0.000–1.600]). Age-stratified data indicated a significant increase in bite incidents among individuals under 15 years old (*p* = 0.027; *τ* = 1.0 [95% CI: 0.200–1.800]), suggesting higher exposure or vulnerability in this age group than in those older than 15 years, who exhibited a non-significant upward trend (*p* = 0.086; *τ* = 0.8 [95% CI: −0.000–1.600]). Dogs were responsible for the majority (63.5%) of bites, with a statistically significant increasing trend (*p* = 0.027; *τ* = 1.0 [95% CI: 0.200–1.800]), whereas cat-related bites accounted for 36.4% and showed a non-significant but increasing trend. Other animal bite sources were rare (0.1%) and did not show a consistent trend. In terms of rabies exposure, Category II bites constituted 73.4% of cases and showed a positive but non-significant trend (*p* = 0.086; *τ* = 0.8 [95% CI: −0.000–1.600]), whereas Category III exposures, accounted for 26.6% and demonstrated variability without a clear monotonic trend (*p* = 0.086; *τ* = 0.8 [95% CI: −0.000–1.600]). To determine whether distinct differences emerged in terms of overall bite incidence, trends were also examined across each congressional district, as shown in Fig. 2.

**Fig. 1:**
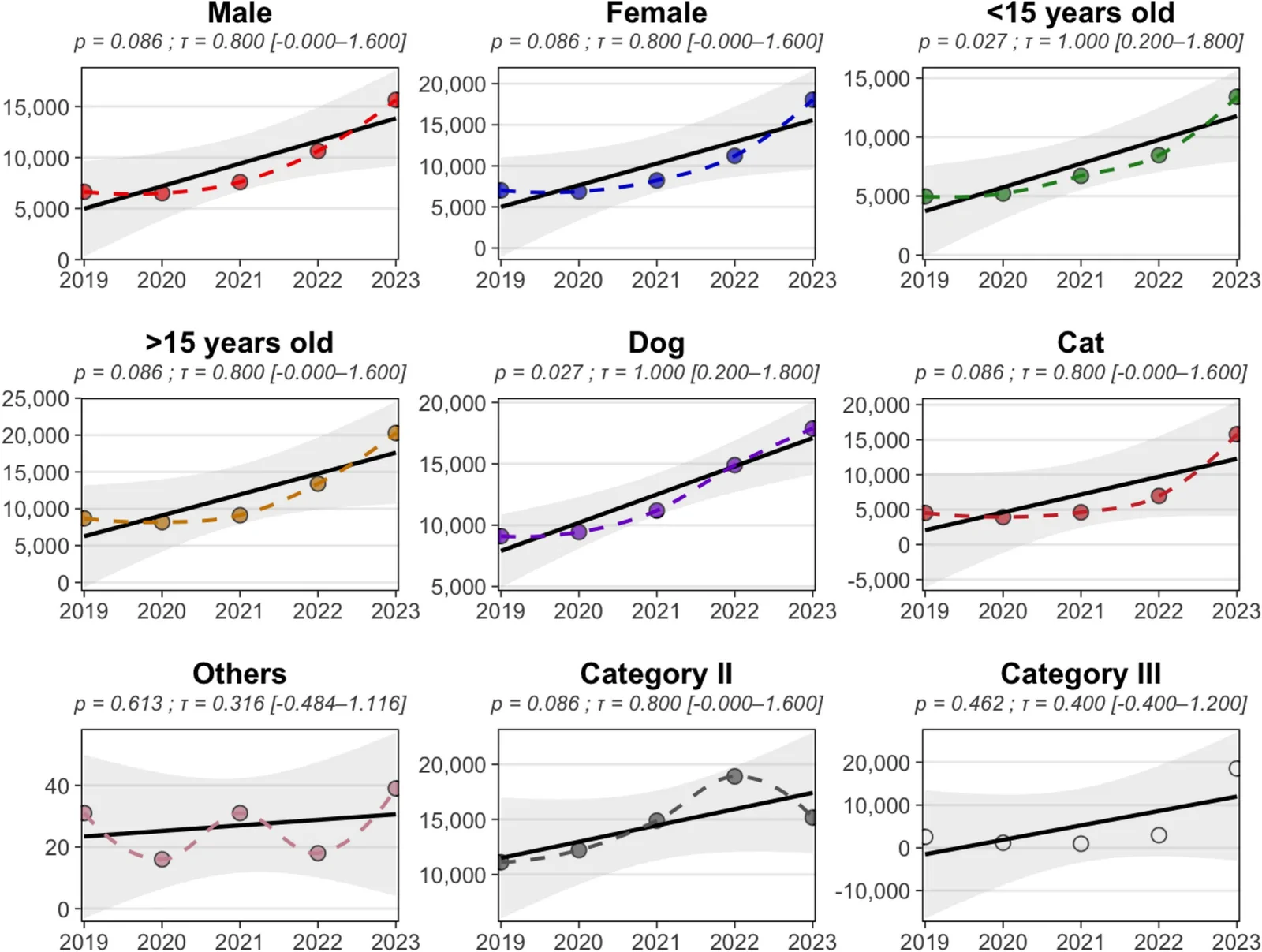
Trend Analysis of Bite Incidents by Demographic and Exposure Characteristics in Davao City, Philippines, 2019–2023. Points represent the observed annual case counts. Solid black lines indicate fitted linear regression trends, with shaded areas representing their 95% confidence intervals. Dashed colored lines represent locally weighted smoothing (LOESS) curves. Reported *p*-values correspond to the Mann–Kendall trend test; Kendall's tau (*τ*) indicates the direction and strength of the monotonic trend. The vertical-axis scales differ between panels.

**Fig. 2:**
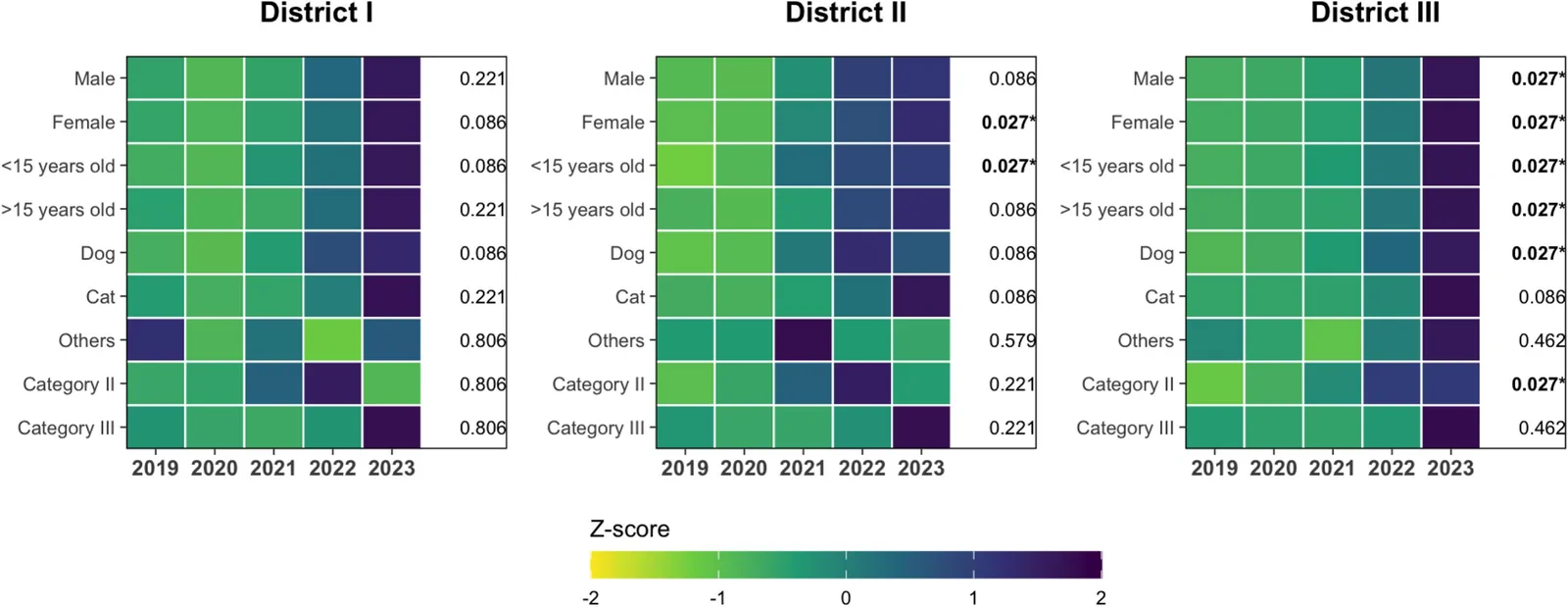
Standardized annual animal-bite counts by demographic and exposure characteristics across Davao City's three congressional districts, 2019–2023. Heatmap colors represent z-scores calculated by subtracting each subgroup's five-year mean annual count within its district from the observed annual count and dividing it by the corresponding sample's standard deviation. Zero indicates the subgroup mean, and positive and negative values indicate above- and below-average counts, respectively. A z-score of +1 indicates a count that is one standard deviation above the mean value. Purple shades represent above-average counts, and green to yellow shades represent below-average counts. Standardization illustrates temporal variations within each subgroup and district, rather than absolute differences in case burden between districts. Values beside each row are Mann–Kendall trend-test *p*-values; bold values with an asterisk indicate *p* < 0.05. (For interpretation of the references to colour in this figure legend, the reader is referred to the web version of this article.)

### 3.2 Distinct differences in temporal trends of animal bite incidents in Davao City, Philippines

Distinct spatial heterogeneity emerged in the temporal trends of animal bite incidents across Davao City's three congressional districts, highlighting the variable One Health dynamics (Fig. 2, Supplementary Table S2). In the 1st district, no statistically significant monotonic trends were observed for bite incidence overall or within demographic and exposure subgroups, indicating stable or non-significant fluctuations over time. Conversely, the 2nd district demonstrated significantly increasing trends in the number of bite incidents among females (*p* = 0.027, *τ* = 1.0 [95% CI: 0.200–1.800]) and individuals under 15 years of age (*p* = 0.027, *τ* = 1.0 [95% CI: 0.200–1.800]), suggesting that these groups experienced notable increases in the number of bite incidents throughout the study period. The 3rd district showed the most robust upward trends, with statistically significant increases across multiple variables, including male and female (both *p* = 0.027, *τ* = 1.0 [95% CI: 0.200–1.800]), age groups below and above 15 years (both *p* = 0.027, *τ* = 1.0 [95% CI: 0.200–1.800]), dog bite incidents (*p* = 0.027, *τ* = 1.0 [95% CI: 0.200–1.800]), and Category II rabies exposures (*p* = 0.027, *τ* = 1.0 [95% CI: 0.200–1.800]). These findings highlight the heterogeneity in the bite incidence dynamics, with the 2nd and 3rd districts showing escalating trends in specific demographics and exposure categories, while the 1st district remained relatively stable.

### 3.3 Trend analysis of bite incidents and rabies mortality by age group, 2019–2022

Given the significant upward trend in bite incidents among individuals under 15 years, further analysis was undertaken to assess whether corresponding trends in rabies-related mortality were present within this age group (Fig. 3, Supplementary Table S3). Although children under 15 years consistently accounted for the majority of rabies-related mortalities (52%), annual data revealed a decreasing pattern in deaths among this population, although the decline was not statistically significant. For individuals older than 15 years, the proportion of rabies deaths was slightly lower (47.8%), and the annual cases remained stable across the study period without evidence of a consistent trend. These findings suggest that despite the increasing incidence of animal bites among children, there was no concurrent statistically significant rise in rabies mortality within this period, underscoring the potential impact of timely post-exposure prophylaxis and rabies control measures.

**Fig. 3:**
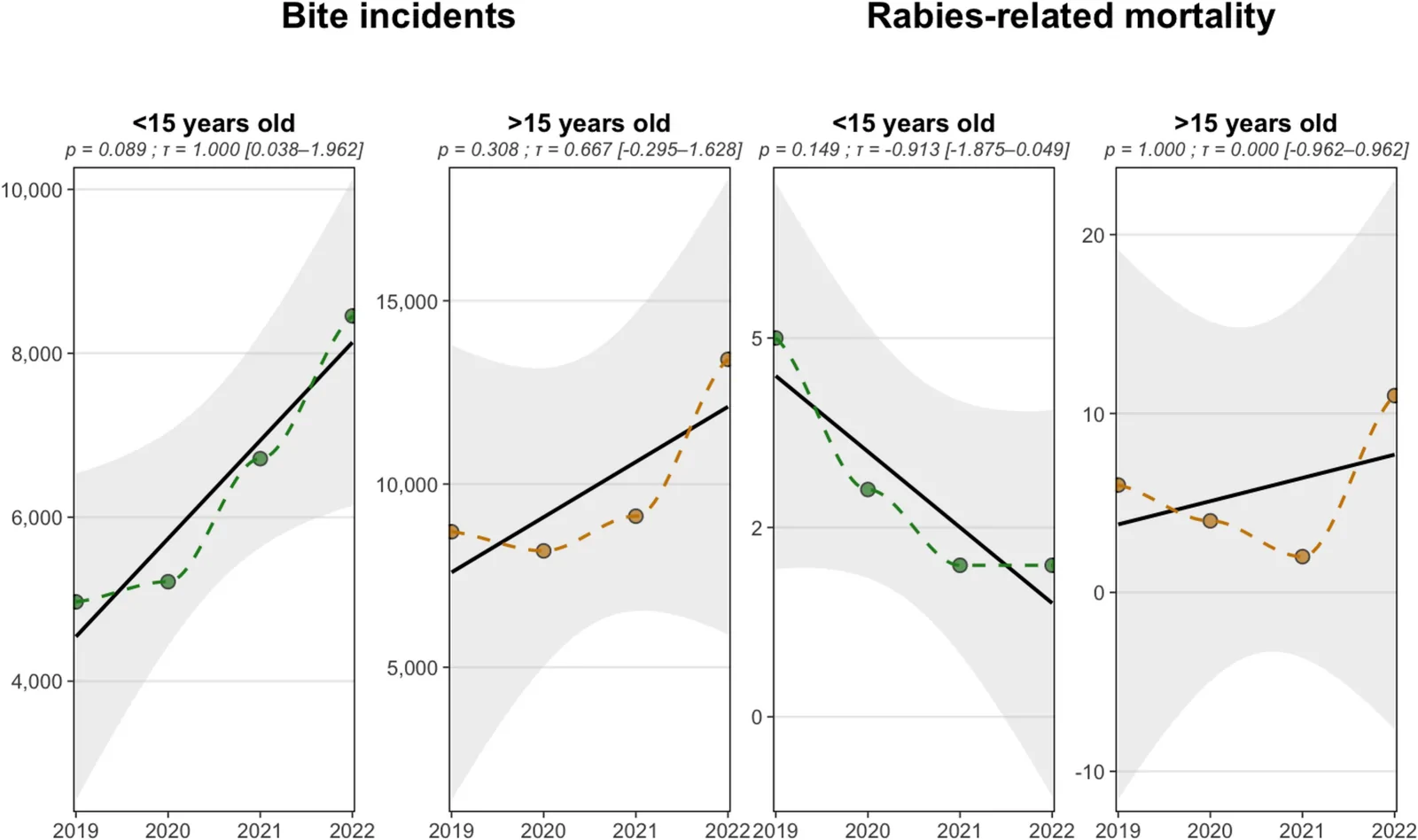
Overall Trend Analysis of Bite Incidents and mortality cases by age in Davao City, Philippines, 2019–2022. Points represent the observed annual case counts. Solid black lines indicate fitted linear regression trends, with shaded areas representing their 95% confidence intervals. Dashed colored lines represent locally weighted smoothing (LOESS) curves. Reported *p*-values correspond to the Mann–Kendall trend test; Kendall's tau (*τ*) indicates the direction and strength of the monotonic trend. The vertical-axis scales differ between panels.

A notable limitation of this analysis is that mortality data were available only by age group and only up to 2022, which precluded a more detailed examination of other critical factors such as sex, geographical district, or exposure category. This limitation highlights the challenges of comprehensive rabies death surveillance and reveals a significant gap in data integration between bite incident reporting systems and mortality registries.

### 3.4 Rabies post-exposure prophylaxis distribution and coverage by exposure category, 2019–2023

Table 1 demonstrates the robust human health sector response within the One Health framework. Tissue culture vaccine (TCV) coverage remained consistently at 100% for all reported Category II and III bite cases throughout the study period, demonstrating equitable vaccine delivery across human exposure in the ABTC network of Davao City. Equine rabies immunoglobulin (ERIG) coverage showed steady improvement from 18.5% in 2019 to 85.9% in 2023 for Category III exposures, reflecting strengthened supply chains critical for comprehensive One Health rabies control. Human rabies immunoglobulin (HRIG) distribution remained minimal prior to 2022 but increased post-pandemic (1.56% in 2022, 1.95% in 2023), indicating partial recovery of immunoglobulin access, although persistent gaps remain.

**Table 1:** Rabies post-exposure prophylaxis distribution and coverage by exposure category in the Davao City, Philippines, 2019–2023.

Year/ Rabies exposure category	Bite cases	TCV		ERIG		HRIG	
		Distributed	coverage^a^	Distributed	coverage^a^	Distributed	coverage^a^
2019							
Category II	11,107	11,107	100				
Category III	2558	2558	100	474	18.53	nd^b^	–
2020							
Category II	12,217	12,217	100				
Category III	1172	1172	100	370	31.57	nd^b^	–
2021							
Category II	14,885	14,885	100				
Category III	952	952	100	563	59.14	nd^b^	–
2022							
Category II	18,908	18,908	100				
Category III	2951	2951	100	2044	69.26	46	1.56
2023							
Category II	15,167	15,167	100				
Category III	18,525	18,525	100	15,906	85.86	361	1.95

a Vaccine coverage (%) was calculated as the proportion of vaccine doses distributed relative to the total number of bite cases within each rabies exposure category per year.

b no vaccine distribution recorded [nd].

## 4 Discussion

Over the five-year period from 2019 to 2023, Davao City recorded a marked increase in reported animal-bite incidents across all age groups and sexes, indicating escalating human exposure to rabies reservoirs at critical urban One Health interface. Children under 15 years accounted for approximately 39.4% of cases, reflecting their greater vulnerability to bites due to close interactions with domestic animals and outdoor activities [13], [14], [15], [16]. This pediatric predominance exemplifies the classic One Health dynamics, where children's behaviors amplify human-animal contact risks. Although no statistically significant trend was observed, a gradual rise in cat bite incidence was noted during the study period. This observation aligns with emerging Asian patterns, where cats represent growing secondary reservoirs, challenging traditional dog-centric rabies control paradigms [2], [17], [18] The City Veterinarian's Office (CVO) has strengthened rabies control through free vaccination and castration services for owned and stray dogs and cats, along with the management of dog pounds and establishment of rehabilitation centers. Despite these veterinary interventions, persistent bite increases highlight gaps in integrated One Health strategies linking animal population control and human surveillance [19], [20].

District-specific heterogeneity further reveals One Health challenges. Even though the citywide incidence of animal bites was primarily observed among children, with dogs as the main source of bites, clear differences emerged in bite incidence trends across Davao City's congressional districts. Remarkably, the 3rd congressional district displayed the most pronounced upward trends across multiple variables (all *p* = 0.027, τ = 1.0), likely reflecting environmental factors such as higher stray densities, uneven veterinary coverage, and population dynamics at district interfaces. The relatively controlled 1st district offers a model for scalable interventions. These spatial patterns underscore the need for geo-targeted One Health coordination between ABTC human surveillance and CVO animal health programs.

The sustained distribution of tissue culture vaccines and increasing provision of ERIG suggest that Davao City's ABTC network maintained an important component of the human health response to animal-bite exposures. However, distribution-based coverage does not ensure timely treatment initiation, vaccination completion, or appropriate immunoglobulin administration, which are essential components of effective PEP [21], [22]. Whitehouse et al. identified deviations from the recommended PEP practices among reported fatal breakthrough infections [23], while Kisaka et al. documented delays in initiating treatment among dog-bite victims in Uganda [24]. Philippine evidence further illustrates this distinction: Amparo et al. reported that 78% of surveyed ABTC patients completed the recommended course, with lack of time and insufficient funds being the main reasons for non-completion [25]. These findings support complementing biological distribution indicators with patient-level monitoring of treatment timeliness and completion. Linking these records would help identify service gaps and guide patient education and follow-up support in Davao City's One Health rabies prevention efforts.

### 4.1 One health gaps and integration opportunities

Although Category III exposures remained stable throughout 2022 despite increasing bite cases, the absence of integrated animal rabies confirmation data represents a critical surveillance gap. ABTCs capture comprehensive human exposure patterns, but CVO-managed animal testing results remain isolated. Linking these datasets would enable risk-stratified PEP, optimize immunoglobulin allocation, and target vaccination campaigns, which are core One Health principles. [17] The rising cat bite trend (36.4% of cases) demands expanded feline control, as urban ecology increasingly positions cats as transmission bridges.

Several methodological limitations should be considered. As a retrospective analysis of human surveillance data, the findings may reflect underreporting and a lack of animal health integration. Coverage reflects distribution rather than confirmed adherence, potentially overestimating its effectiveness. The missing link between ABTC human cases and CVO animal rabies status represents the primary One Health limitation, highlighting opportunities for multisectoral data platforms. Despite these constraints, this study provides valuable urban epidemiology data documenting PEP successes and intervention gaps aligned with the Philippines' 2030 elimination goals.

## 5 Conclusion

This study demonstrates Davao City's resilient One Health implementation through equitable PEP delivery at human-animal interfaces, even amidst pandemic challenges. Sustained progress requires the integration of ABTC surveillance with CVO animal rabies confirmation data, expansion of HRIG access, targeting district-specific escalations, and addressing the rising incidence of cat bites as emerging reservoirs. These multisectoral actions will secure zero human rabies deaths by 2030, positioning Davao City as an urban model for the Philippines' National Rabies Program and the global “Zero by 30” framework.

## CRediT authorship contribution statement

**Alfredo A. Hinay:** Writing – review & editing, Writing – original draft, Visualization, Validation, Supervision, Methodology, Investigation, Formal analysis, Conceptualization. **April Joy Parilla-Sarabia:** Writing – original draft, Methodology, Formal analysis, Data curation. **Jennifer Ashley H. Reyes:** Writing – original draft, Methodology, Investigation, Formal analysis, Data curation. **Mariel Phoebe M. Lopez:** Writing – original draft, Methodology, Formal analysis, Data curation. **Rvin John T. Servillon:** Writing – original draft, Methodology, Investigation.

## Funding

The authors have not declared a specific grant for this research from any funding agency in the public, commercial or not-for-profit sectors.

## Declaration of competing interest

The authors declare that they have no known competing financial interests or personal relationships that could have appeared to influence the work reported in this paper.

## Data Availability

Data are available and will be shared on reasonable request to the corresponding author.

## Appendix A Supplementary data

Supplementary Tables S1–S3. Distribution and temporal trends of animal bite incidents by demographic and exposure characteristics in Davao City and its three congressional districts (2019–2023), and age-specific trends in bite incidents and rabies-related mortality (2019–2022).

## References

[1] Tohma K., Saito M., Kamigaki T., Tuason L.T., Demetria C.S., Orbina J.R.C., Manalo D.L., Miranda M.E., Noguchi A., Inoue S., Suzuki A., Quiambao B.P., Oshitani H. (2014). Phylogeographic analysis of rabies viruses in the Philippines. Infect. Genet. Evol..

[2] Wada Y.A., Mazlan M., Noordin M.M., Mohd-Lila M.A., Fong L.S., Ramanoon S.Z., Zahli N.I.U. (2024). Rabies epidemiology in Malaysia (2015–2023): a cross-sectional insights and strategies for control. Vaccine.

[3] Moore S.M. (2019). Rabies: current preventive strategies. Vet. Clin. North Am. Small Anim. Pract..

[4] Medina D.J.O., Jayme S.I., Amparo A.C.B., Cresencio R.O., Lopez E.L., Baquilod M.S., Hernandez L.M., Villalon E.E.S., Nel L.D. (2016). World rabies day campaign in the Philippines. Trop. Dis. Travel Med. Vaccines.

[5] (2017). Pattern of animal bites and post exposure prophylaxis in rabies in Sri Lanka. Adv. Small Anim. Med. Surg..

[6] Rashid M.M., Chowdhury M.H., Chowdhury M.H.R. (2021). Pattern of animal bites and post exposure prophylaxis for rabies: an one year study in Bangladesh Institute of Tropical Infectious Disease (BITID) Chattogram. Chattagram Maa-O-Shishu Hosp. Med. College J..

[7] Addai J., Nuertey B. (2018). Pattern of animal bite, adherence and delays in initiating post exposure prophylaxis for rabies prevention. Int. J. Infect. Dis..

[8] Quiambao B.P., DyTioco H.Z., Dizon R.M., Crisostomo M.E., Laot T.M., Teuwen D.E. (2008). Rabies post-exposure prophylaxis in the Philippines: health status of patients having received purified equine F(ab)´2 fragment rabies immunoglobulin (favirab). PLoS Negl. Trop. Dis..

[9] Nyasulu P.S., Weyer J., Tschopp R., Mihret A., Aseffa A., Nuvor S.V., Tamuzi J.L., Nyakarahuka L., Helegbe G.K., Ntinginya N.E., Gebreyesus M.T., Doumbia S., Busse R., Drosten C. (2021). Rabies mortality and morbidity associated with animal bites in Africa: a case for integrated rabies disease surveillance, prevention and control: a scoping review. BMJ Open.

[10] Yan S., Chen Y., Ye W., Chen F., Li L. (2019). Characteristics and factors associated with post-exposure prophylaxis (PEP) treatment of dog and cat bites among left-behind children: a cross-sectional study in two cities of China. BMJ Open.

[11] United Against Rabies Collaboration (2018).

[12] Valenzuela L.M., Jayme S.I., Amparo A.C.B., Taylor L.H., Cruz M.P.Z.D., Licuan D.A., Gamal-Bitao R., Nel L.H. (2017). The Ilocos Norte communities against rabies exposure elimination project in the Philippines: epidemiological and economic aspects. Front. Vet. Sci..

[13] Sriaroon C., Sriaroon P., Daviratanasilpa S., Khawplod P., Wilde H. (2006). Retrospective: animal attacks and rabies exposures in Thai children. Travel Med. Infect. Dis..

[14] Ramos J.M., Melendez N., Reyes F., Gudiso G., Biru D., Fano G., Aberra G., Tessema D., Tesfamariam A., Balcha S., Gutiérrez F. (2015). Epidemiology of animal bites and other potential rabies exposures and anti-rabies vaccine utilization in a rural area in Southern Ethiopia. Ann. Agric. Environ. Med..

[15] Marrana M. (2022). One Health.

[16] Gouin G.G., Aenishaenslin C., Lévesque F., Simon A., Ravel A. (2021). Description and determinants of at-risk interactions for human health between children and dogs in an Inuit Village. Anthrozoos.

[17] Nguyen V.L., Dantas-Torres F., Otranto D. (2021). Canine and feline vector-borne diseases of zoonotic concern in Southeast Asia. Curr. Res. Parasitol. Vector-Borne Dis..

[18] Piyaphanee W., Shantavasinkul P., Phumratanaprapin W., Udomchaisakul P., Wichianprasat P., Benjavongkulchai M., Ponam T., Tantawichian T. (2010). Rabies exposure risk among foreign backpackers in Southeast Asia. Am. J. Trop. Med. Hyg..

[19] Diamante E., Herrada N., Lachica Z.P., Oguis G.F., Alviola P., Mata M.A. (2021). Cost optimization of the intensified rabies control program in Davao City, Philippines using linear programming. Philipp. J. Sci..

[20] Janeaim N., Suwanwong C., Prasittichok P., Mohan K.P., Hudrudchai S. (2024). Effectiveness of educational interventions for improving rabies prevention in children: a systematic review and meta-analysis. J. Educ. Health Promot..

[21] Nadal D., Bote K., Masthi R., Narayana A., Ross Y., Wallace R., Abela B. (2023). Rabies post-exposure prophylaxis delivery to ensure treatment efficacy and increase compliance. IJID One Health.

[22] Kaboré D.S., Tranchot-Diallo J., Savadogo M., Zoungrana J., Kaboré N.F., Millogo A., Ouédraogo A.-S., Hien H. (2026). Completion of rabies post-exposure prophylaxis in Ouagadougou, Burkina Faso, 2021–2023: a cross-sectional analysis of routine data. PLoS Negl. Trop. Dis..

[23] Whitehouse E.R., Mandra A., Bonwitt J., Beasley E.A., Taliano J., Rao A.K. (2023). Human rabies despite post-exposure prophylaxis: a systematic review of fatal breakthrough infections after zoonotic exposures. Lancet Infect. Dis..

[24] Kisaka S., Makumbi F., Majalija S., Bahizi G., Thumbi S. (2022). Delays in initiating rabies post-exposure prophylaxis among dog bite victims in Wakiso and Kampala districts, Uganda. Open Res. Afr..

[25] Amparo A.C.B., Jayme S.I., Roces M.C.R., Quizon M.C.L., Mercado M.L.L., Dela Cruz M.P.Z., Licuan D.A., Villalon E.E.S., Baquilod M.S., Hernandez L.M., Taylor L.H., Nel L.H. (2018). The evaluation of animal bite treatment centers in the Philippines from a patient perspective. PLoS One.

