# Supplementary material for: Epidemiology of animal bites and post-exposure vaccination coverage in Davao City, Philippines: A five-year retrospective cohort study, 2019–2023
Source: One Health. 2026 Oct 17;23:101622. doi: 10.1016/j.onehlt.2026.101622 (PMC13697316; doi:10.1016/j.onehlt.2026.101622)
Supplement: Supplementary file 1 [file mmc1.docx]

**Supplementary Table S1.** Distribution and Trend Analysis of Bite Incidents by Demographic and Exposure Characteristics in Davao City, Philippines, 2019–2023

|  | Overall | 2019 | 2020 | 2021 | 2022 | 2023 | Mann-Kendall test | |  |
| --- | --- | --- | --- | --- | --- | --- | --- | --- | --- |
|  | n (%) | n (%) | n (%) | n (%) | n (%) | n (%) | *p* | *τ* [95 CI] | |
| Sex |  |  |  |  |  |  |  |  | |
| Male | 47,030 (47.8%) | 6645 (14.13%) | 6501 (13.82%) | 7599 (16.16%) | 10638 (22.62%) | 15647 (33.27%) | 0.086 | 0.800 [-0.000 – 1.600] | |
| Female | 51,412 (52.2%) | 7020 (13.65%) | 6888 (13.4%) | 8238 (16.02%) | 11221 (21.83%) | 18045 (35.1%) | 0.086 | 0.800 [-0.000 – 1.600] | |
| Age |  |  |  |  |  |  |  |  | |
| <15 years old | 38,767 (39.4%) | 4968 (12.82%) | 5214 (13.45%) | 6715 (17.32%) | 8456 (21.81%) | 13414 (34.6%) | **0.027** | 1.000 [0.200 – 1.800] | |
| >15 years old | 59,675 (60.6%) | 8697 (14.57%) | 8175 (13.7%) | 9122 (15.29%) | 13403 (22.46%) | 20278 (33.98%) | 0.086 | 0.800 [-0.000 – 1.600] | |
| Bite source |  |  |  |  |  |  |  |  | |
| Dog | 62,489 (63.5%) | 9102 (14.57%) | 9436 (15.1%) | 11191 (17.91%) | 14886 (23.82%) | 17874 (28.6%) | **0.027** | 1.000 [0.200 – 1.800] | |
| Cat | 35,818 (36.4%) | 4532 (12.65%) | 3937 (10.99%) | 4615 (12.88%) | 6955 (19.42%) | 15779 (44.05%) | 0.086 | 0.800 [-0.000 – 1.600] | |
| Others | 135 (0.1%) | 31 (22.96%) | 16 (11.85%) | 31 (22.96%) | 18 (13.33%) | 39 (28.89%) | 0.613 | 0.316 [-0.484 – 1.116] | |
| Rabies exposure category | |  |  |  |  |  |  |  | |
| Category II | 72,284 (73.4%) | 11107 (15.37%) | 12217 (16.9%) | 14885 (20.59%) | 18908 (26.16%) | 15167 (20.98%) | 0.086 | 0.800 [-0.000 – 1.600] | |
| Category III | 26,158 (26.6%) | 2558 (9.78%) | 1172 (4.48%) | 952 (3.64%) | 2951 (11.28%) | 18525 (70.82%) | 0.462 | 0.400 [-0.400 – 1.200] | |

**Supplementary Table S2.** Distribution and Trend Analysis of Bite Incidents by Demographic and Exposure Characteristics in Three Congressional Districts, 2019–2023

|  | Overall | 2019 | 2020 | 2021 | 2022 | 2023 | Mann-Kendall test | |  |  |
| --- | --- | --- | --- | --- | --- | --- | --- | --- | --- | --- |
|  | n (%) | n (%) | n (%) | n (%) | n (%) | n (%) | *p* | *τ* [95 CI] | |  |
| 1^st^ congressional district | |  |  |  |  |  |  |  | |  |
| Sex |  |  |  |  |  |  |  |  | |  |
| Male | 19670 (46.8%) | 3345 (17%) | 2973 (15.1%) | 3337 (17%) | 4329 (22%) | 5686 (28.9%) | 0.221 | 0.600 [-0.200 – 1.400] | |  |
| Female | 22349 (53.2%) | 3658 (16.4%) | 3293 (14.7%) | 3731 (16.7%) | 4789 (21.4%) | 6878 (30.8%) | 0.086 | 0.800 [-0.000 – 1.600] | |  |
| Age |  |  |  |  |  |  |  |  | |  |
| <15 years old | 15245 (36.3%) | 2462 (16.1%) | 2312 (15.2%) | 2797 (18.3%) | 3251 (21.3%) | 4423 (29%) | 0.086 | 0.800 [-0.000 – 1.600] | |  |
| >15 years old | 26774 (63.7%) | 4541 (17%) | 3954 (14.8%) | 4271 (16%) | 5867 (21.9%) | 8141 (30.4%) | 0.221 | 0.600 [-0.200 – 1.400] | |  |
| Bite source |  |  |  |  |  |  |  |  | |  |
| Dog | 25615 (61%) | 4430 (17.3%) | 4253 (16.6%) | 4734 (18.5%) | 5823 (22.7%) | 6375 (24.9%) | 0.086 | 0.600 [-0.200 – 1.400] | |  |
| Cat | 16365 (38.9%) | 2558 (15.6%) | 2010 (12.3%) | 2325 (14.2%) | 3294 (20.1%) | 6178 (37.8%) | 0.221 | 0.600 [-0.200 – 1.400] | |  |
| Others | 39 (0.1%) | 15 (38.5%) | 3 (7.7%) | 9 (23.1%) | 1 (2.6%) | 11 (28.2%) | 0.806 | -2 [-1.000 – 0.600] | |  |
| Rabies exposure category | |  |  |  |  |  |  |  | |  |
| Category II | 31257 (46.8%) | 5662 (18.1%) | 5725 (18.3%) | 6703 (21.4%) | 7778 (24.9%) | 5389 (17.2%) | 0.806 | 0.200 [-0.600 – 1.000] | |  |
| Category III | 10762 (53.2%) | 1341 (12.5%) | 541 (5%) | 365 (3.4%) | 1340 (12.5%) | 7175 (66.7%) | 0.806 | 0.200 [-0.600 – 1.000] | |  |
| 2^nd^ congressional district | |  |  |  |  |  |  |  | |  |
| Sex |  |  |  |  |  |  |  |  | |  |
| Male | 8154 (47.5%) | 1166 (14.3%) | 1156 (14.2%) | 1503 (18.4%) | 2116 (26%) | 2213 (27.1%) | 0.086 | 0.800 [0.000 – 1.600] | |  |
| Female | 9011 (52.5%) | 1230 (13.6%) | 1268 (14.1%) | 1731 (19.2%) | 2218 (24.6%) | 2564 (28.5%) | **0.027** | **1.000 [0.200 – 1.800]** | |  |
| Age |  |  |  |  |  |  |  |  | |  |
| <15 years old | 6601 (38.5%) | 827 (12.5%) | 970 (14.7%) | 1437 (21.8%) | 1642 (24.9%) | 1725 (26.1%) | **0.027** | **1.000 [0.200 – 1.800]** | |  |
| >15 years old | 10564 (61.5%) | 1569 (14.9%) | 1454 (13.8%) | 1797 (17%) | 2692 (25.5%) | 3052 (28.9%) | 0.086 | 0.800 [0.000 – 1.600] | |  |
| Bite source |  |  |  |  |  |  |  |  | |  |
| Dog | 11050 (64.4%) | 1595 (14.4%) | 1665 (15.1%) | 2266 (20.5%) | 2973 (26.9%) | 2551 (23.1%) | 0.086 | 0.800 [0.000 – 1.600] | |  |
| Cat | 6068 (35.4%) | 794 (13.1%) | 752 (12.4%) | 948 (15.6%) | 1354 (22.3%) | 2220 (36.6%) | 0.086 | 0.800 [0.000 – 1.600] | |  |
| Others | 47 (0.3%) | 7 (14.9%) | 7 (14.9%) | 20 (42.6%) | 7 (14.9%) | 6 (12.8%) | 0.579 | 0.400 [-1.159 – 0.442] | |  |
| Rabies exposure category | |  |  |  |  |  |  |  | |  |
| Category II | 13204 (46.8%) | 1906 (14.4%) | 2200 (16.7%) | 2985 (22.6%) | 3786 (28.7%) | 2327 (17.6%) | 0.221 | 0.600 [-0.200 – 1.400] | |  |
| Category III | 3961 (53.2%) | 490 (12.4%) | 224 (5.7%) | 249 (6.3%) | 548 (13.8%) | 2450 (61.9%) | 0.221 | 0.600 [-0.200 – 1.400] | |  |
| 3^rd^ congressional district | |  |  |  |  |  |  |  | |  |
| Sex |  |  |  |  |  |  |  |  | |  |
| Male | 19206 (48.9%) | 2134 (11.1%) | 2372 (12.4%) | 2759 (14.4%) | 4193 (21.8%) | 7748 (40.3%) | **0.027** | **1.000 [0.200 – 1.800]** | |  |
| Female | 20052 (51.1%) | 2132 (10.6%) | 2327 (11.6%) | 2776 (13.8%) | 4214 (21%) | 8603 (42.9%) | **0.027** | **1.000 [0.200 – 1.800]** | |  |
| Age |  |  |  |  |  |  |  |  | |  |
| <15 years old | 16921 (43.1%) | 1679 (9.9%) | 1932 (11.4%) | 2481 (14.7%) | 3563 (21.1%) | 7266 (42.9%) | **0.027** | **1.000 [0.200 – 1.800]** | |  |
| >15 years old | 22337 (56.9%) | 2587 (11.6%) | 2767 (12.4%) | 3054 (13.7%) | 4844 (21.7%) | 9085 (40.7%) | **0.027** | **1.000 [0.200 – 1.800]** | |  |
| Bite source |  |  |  |  |  |  |  |  | |  |
| Dog | 25824 (65.8%) | 3077 (11.9%) | 3518 (13.6%) | 4191 (16.2%) | 6090 (23.6%) | 8948 (34.6%) | **0.027** | **1.000 [0.200 – 1.800]** | |  |
| Cat | 13385 (34.1%) | 1180 (8.8%) | 1175 (8.8%) | 1342 (10%) | 2307 (17.2%) | 7381 (55.1%) | 0.086 | 0.800 [-0.000 – 1.600] | |  |
| Others | 49 (0.1%) | 9 (18.4%) | 6 (12.2%) | 2 (4.1%) | 10 (20.4%) | 22 (44.9%) | 0.462 | 0.400 [-0.400 – 1.200] | |  |
| Rabies exposure category | |  |  |  |  |  |  |  | |  |
| Category II | 27823 (70.9%) | 3539 (12.7%) | 4292 (15.4%) | 5197 (18.7%) | 7344 (26.4%) | 7451 (26.8%) | **0.027** | **1.000 [0.200 – 1.800]** | |  |
| Category III | 11435 (29.1%) | 727 (6.4%) | 407 (3.6%) | 338 (3%) | 1063 (9.3%) | 8900 (77.8%) | 0.462 | 0.400 [-0.400 – 1.200] | |  |

**Supplementary Table S3.** Overall Trend Analysis of Bite Incidents and mortality cases by age in Davao City, Philippines, 2019–2022

|  | Overall | 2019 | 2020 | 2021 | 2022 | Mann-Kendall test | |
| --- | --- | --- | --- | --- | --- | --- | --- |
|  | n (%) | n (%) | n (%) | n (%) | n (%) | *p* | τ [95 CI] |
| Bite incidents |  |  |  |  |  |  |  |
| <15 years old | 25,353 (39.2%) | 4968 (34.8%) | 5214 (36.8%) | 6715 (44.4%) | 8456 (46.0%) | 0.089 | 0.900 [0.038 – 1.962] |
| >15 years old | 39,397 (60.8%) | 8697 (61.0%) | 8175 (57.7%) | 9122 (60.3%) | 13403 (58.0%) | 0.308 | 0.600 [-0.295 – 1.628] |
| Rabies-related mortality | |  |  |  |  |  |  |
| <15 years old | 12 (52.2%) | 5 (41.7%) | 3 (25.0%) | 2 (16.7%) | 2 (16.7%) | 0.149 | -0.900 [-1.875 – 0.049] |
| >15 years old | 23 (47.8%) | 6 (26.1%) | 4 (17.4%) | 2 (8.7%) | 11 (47.8%) | 1.0 | 0.000 [-0.962 – 0.962] |
